# Enhanced zero-phonon line emission from an ensemble of W centers in circular and bowtie Bragg grating cavities

**DOI:** 10.1515/nanoph-2024-0485

**Published:** 2024-11-19

**Authors:** Vijin Kizhake Veetil, Junyeob Song, Pradeep N. Namboodiri, Nikki Ebadollahi, Ashish Chanana, Aaron M. Katzenmeyer, Christian Pederson, Joshua M. Pomeroy, Jeffrey Chiles, Jeffrey Shainline, Kartik Srinivasan, Marcelo Davanco, Matthew Pelton

**Affiliations:** Department of Physics, 14701 University of Maryland Baltimore County, 1000 Hilltop Circle, Baltimore, MD 21250, USA; National Institute of Standards and Technology, 100 Bureau Dr, Gaithersburg, MD 20899, USA; Theiss Research, La Jolla, CA 92073, USA; University of Maryland College Park, 4500 Campus Dr, College Park, MD 20742, USA; National Institute of Standards and Technology, 325 Broadway, Boulder, CO 80305, USA; Joint Quantum Institute, 4254 Stadium Dr, College Park, MD 20742, USA

**Keywords:** quantum emitter, color center, silicon defect, Purcell factor, enhanced emission

## Abstract

Color centers in silicon have recently gained considerable attention as single-photon sources and as spin qubit-photon interfaces. However, one of the major bottlenecks to the application of silicon color centers is their low overall brightness due to a relatively slow emission rate and poor light extraction from silicon. Here, we increase the photon collection efficiency from an ensemble of a particular kind of color center, known as W centers, by embedding them in circular Bragg grating cavities resonant with their zero-phonon-line emission. We observe a ≈5-fold enhancement in the photon collection efficiency (the fraction of photons extracted from the sample and coupled into a single-mode fiber), corresponding to an estimated ≈11-fold enhancement in the photon extraction efficiency (the fraction of photons collected by the first lens above the sample). For these cavities, we observe lifetime reduction by a factor of ≈1.3 . For W centers in resonant bowtie-shaped cavities, we observed a ≈3-fold enhancement in the photon collection efficiency, corresponding to a ≈6-fold enhancement in the photon extraction efficiency, and observed a lifetime reduction factor of ≈1.1 . The bowtie cavities thus preserve photon collection efficiency and Purcell enhancement comparable to circular cavities while providing the potential for utilizing in-plane excitation methods to develop a compact on-chip light source.

## 1 Introduction

Silicon not only remains the basis of nearly all electronic integrated circuits but is also emerging as the platform of choice for integrated photonics [1], [2], [3]. However, one of the main bottlenecks silicon photonics applications face is the lack of an efficient on-chip light source, although there have been significant improvements in demonstrating both classical [4], [5], [6], [7], [8] and quantum [9], [10] light sources. Color centers in silicon have recently emerged as a viable candidate for quantum information applications, both as single-photon sources and for interfacing stationary qubits with photons, essential components for quantum networking and multiprocessor quantum computing [11]. Color centers in silicon have been studied since at least the 1980s [12], [13], [14], but only recently have bright single-photon telecommunications band emissions been shown [15], [16], [17], [18].

One of the main challenges while working with silicon defect centers is the extraction of emission from the semiconductor due to the high refractive index of silicon [19]. For luminescent defects in silicon to serve as useful single-photon sources for quantum photonics, emitted photons must be efficiently extracted from the silicon host and funneled into a useful optical spatial mode (e.g., a low-diffraction Gaussian beam or an on-chip waveguide mode). This can be achieved by leveraging cavity quantum electrodynamics (cQED) effects, where the emitter is placed in a microscopic optical cavity designed to support optical resonances. This enables the emitters in resonance with the cavity mode to efficiently radiate light to desirable free-space or guided optical modes [20]. In addition to enhanced source efficiency, the enhancement of the radiative rate by the Purcell effect can be leveraged to reach higher single photon emission rates [21], [22], [23], [24] and to bring the emitted photons closer to the Fourier transform limit, for improved photon indistinguishability [25], [26]. The Purcell effect can also potentially be leveraged for color centers to enhance the decay rate into the zero-phonon line (ZPL) transition, leading to an improved Debye–Waller (DW) factor and enhanced quantum efficiency (QE) [27], [28].

The first color center to be studied specifically for quantum-information applications was the G center, which consists of an interstitial silicon atom surrounded by two substitutional carbon atoms. Single G centers were optically isolated and were shown to act as stable, bright, efficient sources of single photons [18], [29], [30]. Improved light extraction from an ensemble of G centers has been demonstrated by incorporating them in micropillars [31], microrings [32], and Mie resonators [33]. 2D photonic crystal cavities have been employed to enhance ZPL emission of single G centers with an 8-fold decay acceleration [34]. T centers are radiation damage centers in silicon that consist of two carbon atoms, one hydrogen atom, and an unpaired electron. The presence of a spin ground state makes them suitable candidates for optically active spin qubits with millisecond coherence time [35]. However, the relatively long lifetime of T centers, ≈1 μs, limits their brightness. Cavity-enhanced emission has been demonstrated for a single T center in micropuck [16], waveguide [36], and photonic crystal structures [37], [38].

G centers and T centers have relatively low fractions of ZPL emission (DW ≈ 15 % and ≈23 %, respectively). By contrast, W centers, native defects consisting of a tri-interstitial Si complex [39], offer higher DW factors of ≈40 % [17]. Single W centers have been isolated optically and used to generate single photons. W centers also have been a choice of quantum emitter toward building an on-chip silicon light source, and some of the attempts include embedding them in a microring [40] and circular Bragg grating structures [41]. Buckley et al. demonstrated waveguide-coupled Si light-emitting diodes by electrically pumping the W centers implanted in the intrinsic region of a p-i-n diode and using waveguide-coupled superconducting nanowires single photon detectors as the receivers to create an on-chip optical link [8].

In this work, we investigate cQED effects in circular Bragg grating (CBG) cavities, commonly known as bullseye cavities, fabricated on silicon-on-insulator (SOI) material containing ensembles of W centers. CBG cavities have been widely used in cQED experiments because they offer both high extraction efficiency into a near-Gaussian free-space beam with relatively small divergence as well as a modest Purcell radiative rate enhancement [42], [43], [44], [45], [46], [47], [48], [49]. Bullseye cavities also offer a relatively broad extraction bandwidth, simplifying spectral overlap with the quantum emitters.

**Figure 1:**
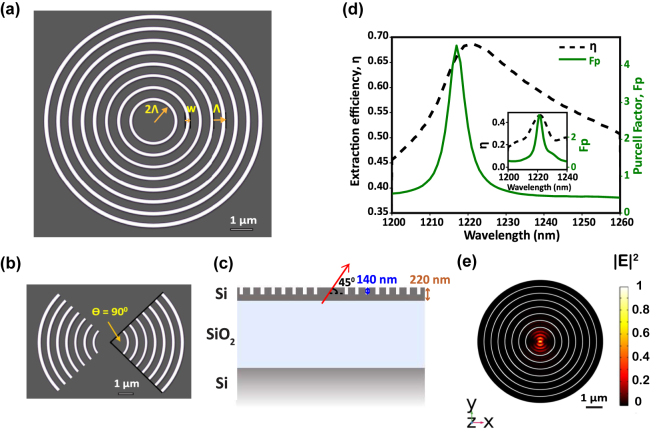
A single W center dipole in the center of a Bragg grating cavity. (a, b) Top-view of circular Bragg grating (CBG) and bowtie grating cavities. White circles represent the etched trenches, and the gray region represents unetched silicon (a) schematic of a CBG (bullseye) cavity with seven rings. 2Λ is the central disk radius, w is the trench width, and Λ is the grating period. (b) Schematic of a bowtie cavity, with a cavity enclosure angle of 90°. (c) Schematic of the cross-sectional view of a partially etched bullseye cavity on SOI with a trench depth of 140 nm and a silicon device layer thickness of 220 nm. The red arrow represents a W center dipole pointed in the [111] direction at 45° with respect to the cavity plane. (d) Finite Element Method (FEM) simulation of a W center dipole placed in the center of a bullseye cavity at 45° with respect to the cavity plane. Plot showing the Purcell factor (*F*_p_) and extraction efficiency (*η*) into a 0.9 numerical aperture (NA) objective for a dipole placed in the center of a partially etched bullseye cavity resonant at ZPL. The inset shows the same curves for a 90° h-bowtie cavity, with the dipole oriented perpendicular to the horizontal axis of the cavity and at 45° with respect to the cavity plane. (e) Normalized electric field intensity of the resonant mode in the XY plane. Black rings represent the circular gratings, and the white circles represent the etched trenches of the bullseye cavity.

We report an enhancement of ZPL photoluminescence (PL) collection into a microscope objective by a factor of ≈11, as well as radiative decay-rate enhancement by a factor of ≈1.3, for an ensemble of W centers embedded in a ZPL-resonant bullseye cavity. Contrasting with Le Faucher et al. [41] and Chiles and Shainline [50], here we investigate the effect of two different geometrical variations that provide advantages in both performance and application. Specifically, we investigate cavities with 1) bowtie shape and 2) partially etched trenches as shown in Figure 1. For bowtie-shaped cavities resonant with the ZPL, we report a radiative rate enhancement of ≈1.1 and a ZPL collection enhancement of ≈6 times in the first lens (≈3 times into SMF-28 optical fiber). Bowtie cavities provide straightforward, direct optical and electrical access to embedded color centers from their open ends and, therefore, may provide advantages in devices where capabilities are desired such as current injection or waveguided resonant optical excitation. Moreover, employing a partial etching strategy improves extraction efficiency and helps mitigate the effects of power-induced thermal heating, which has been shown to affect the recombination processes of W centers embedded in a fully etched bullseye cavity [41].

## 2 Cavity design and optimization

We designed partially etched circular Bragg grating cavities, shown in Figure 1(a), and bowtie cavities, schematically displayed in the Figure 1(b), to investigate Purcell enhancement and improved extraction efficiency. Partial etching increases extraction efficiency due to the vertical asymmetry of the geometry, which favors upward light scattering [42]. From Finite Element Method (FEM) simulations, the cavity parameters are optimized to enhance the vertical light extraction efficiency (see Figure S1(A and B) in the Supplementary information). Starting with a total thickness of 220 nm for the silicon device layer, the optimized cavity parameters are Λ = 440 nm (pitch), *w* = 90 nm (trench width), *b* = 350 nm (grating width), and *t*_trench_ = 140 nm (trench depth).

Figure 1(c) shows the schematic of the vertical stack of SOI with a partially etched grating structure in the device layer. In the simulation, an electric point dipole emulating a single W center is placed in the center of this particular cavity design at 45° with respect to the cavity plane (the W center dipole moment is oriented along [111], and the cavity is on the [100] wafer plane), resulting in a maximum Purcell factor, *F*_p_, of 4.5 and extraction efficiency, *η*, of 65 %, as displayed in Figure 1(d). The Purcell factor, *F*_p_, is calculated by taking the ratio of the total power radiated into the environment by a dipole in the resonant cavity to the total power radiated by a dipole in homogenous silicon, and the extraction efficiency, *η*, is calculated by taking the ratio of the total power radiated through a parametric surface emulating a 0.9 numerical aperture (NA) objective to the total power radiated into all directions. The Purcell factor curve in Figure 1(d) is fitted with a Lorentzian to determine the simulated quality factor (*Q*) of the cavity of 203 ± 1.01 (all uncertainties are 95 % confidence intervals. Details are in the Supplementary information).

We performed the same simulation for a bowtie cavity with a cavity enclosure angle *θ* = 90°, with the dipole oriented perpendicular to the horizontal axis of the cavity and at 45° with respect to the cavity plane. We obtain *F*_p_ ≈ 3.5 and *η* ≈ 40 %, as shown in the inset of Figure 1(d). By fitting a Lorentzian function to the simulated Purcell factor curve, we obtain a *Q* of 183 ± 2.12 for the bowtie cavity. The bowtie structure has a lower *Q* due to the open ends of the cavity, where the mode is no longer strictly confined. According to simulations, an open cavity with an enclosure angle of 140° would further improve the extraction efficiency and the Purcell factor (see Figure S2(A and B) in the Supplementary information).

Polarization-resolved PL measurements [29] indicate that W centers emit light polarized along two crystal axes orientations, [110] and [1 $\bar{1}$ 0 ]. Figure 1(e) shows the electric field intensity of the in-plane cavity transverse electric (TE) mode for a dipole oriented along the *x* direction, which corresponds to one of the crystal axes’ orientations of the W centers. Due to the circular symmetry of the cavities, the same mode profile is expected for a dipole placed in the center of the cavity oriented in the *y* direction. Therefore, an unpolarized far-field emission is expected for an ensemble of emitters with random orientations in the bullseye cavity. Bowtie cavities, on the other hand, support only one mode to which the dipole couples, leading to polarized far-field emission for an ensemble [51].

## 3 Sample and cavity fabrication

We implanted carbon in a (100)-oriented silicon-on-insulator (SOI) wafer with a device layer thickness of 220 nm and a buried oxide thickness of 3 μm. The ion implantation voltage was set to 30 keV, with a fluence of 5 × 10^11^ cm^−2^, to achieve a mean ion depth of 100 nm into the device layer, according to Stopping and Range of Ions in Matter (SRIM) simulations [52] (see Figure S3 in the Supplementary information). The wafer was then cleaved into 10 mm × 10 mm dies, and selected dies were annealed at 250 °C for 60 s to create an ensemble of W centers. This annealing step is crucial as it increases the signal-to-background ratio by repairing lattice damage caused by the implantation process and suppressing background emission [53].

**Figure 2:**
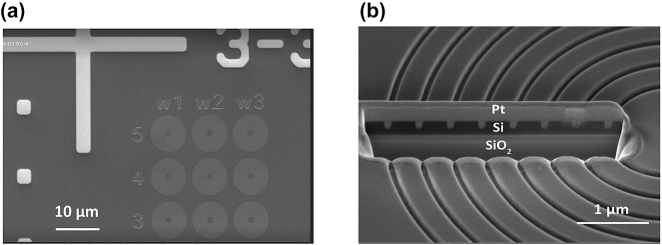
Fabricated partially etched circular Bragg grating (bullseye) cavities containing W centers. (a) Top view of the scanning electron micrograph (SEM) of bullseye cavities with varying parameters. (b) SEM of a bullseye cavity cross section, showing seven partially etched rings around the central disk. The cross section was created by a focused ion beam (FIB), where platinum (Pt) was deposited before ion milling to reduce charging effects.

Subsequently, we use electron-beam lithography and inductively coupled plasma reactive ion etching (ICP-RIE) to fabricate dielectric cavities (see Section 3 of the Supplementary information for details). Cavities were produced as shown in Figure 2(a), with varying parameters to produce resonances at varying spectral detuning from the W center ZPL frequency. A scanning electron micrograph (SEM) of the bullseye cavity after partial etching is shown in Figure 2(b).

## 4 Experimental methods

Figure S4 in the Supplementary information presents a schematic of the experimental setup used for the optical measurements. A 635 nm continuous-wave (CW) laser or a 640 nm pulsed laser was used to excite the W centers in silicon. The samples were mounted on a piezo-positioner stack in a cryostat operating at 10 K. We used a 0.9 NA objective to focus the laser beam to a nearly diffraction-limited spot on the sample surface. The output photoluminescence (PL) from the W centers was collected by the same objective and coupled to a single-mode fiber connected to the entrance slit of a spectrometer. For all the measurements taken, a 900 nm long pass filter and a 1,100 nm long pass filter blocked the laser light and other residual background emission from silicon. A halogen lamp source was used to image the sample surface, including cavities, and to position the excitation laser on the sample. A short-wave infrared camera was also used to perform near-field imaging of the PL from the cavities under CW excitation. The power-dependent PL studies and lifetime measurements were performed by exciting the color centers with a pulsed laser and collecting only the ZPL photons with the help of a tunable bandpass filter with a center wavelength of 1,218 nm and a bandwidth of 1 nm. The filtered ZPL emission was fiber-coupled and sent to a superconducting nanowire single photon detector (SNSPD), which was connected to a time tagger module to register photon arrival time for time-resolved PL measurements. The CBG cavity resonance was measured by sending a supercontinuum laser (400–2000 nm) into the cavity through a fiber circulator and redirecting the reflected laser to an optical spectrum analyzer.

## 5 Results

### 5.1 Measurement of enhanced collection efficiency

**Figure 3:**
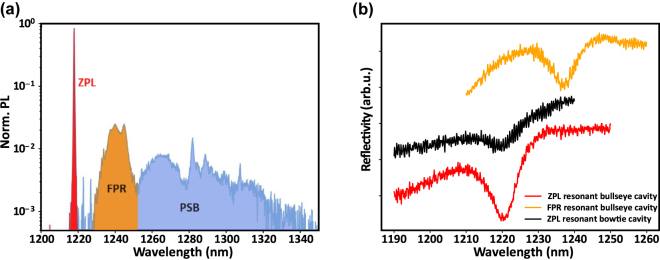
Different segments in the W center PL spectrum and their associated resonant cavities. (a) Photoluminescence spectrum of a W center ensemble in silicon on insulator at 10 K. ZPL: zero-phonon line. FPR: first phonon replica. PSB: phonon sideband. (b) Supercontinuum laser reflectivity spectra of bullseye cavity resonant with the ZPL (red), bowtie cavity resonant with the ZPL (black), and bullseye cavity resonant with the FPR (orange).

Figure 3(a) shows the emission spectrum of W centers, which consists of intense zero-phonon line (ZPL) emission at ≈1.018 eV (1,218 nm) and a broad phonon sideband (PSB) at lower energy due to phonon-assisted recombination processes. The first phonon replica (FPR) accounts for a large fraction of the sideband emission. The fraction of photons emitted into the ZPL over the total emission in the range shown in Figure 3(a) is ≈30% , somewhat smaller than the ≈40% Debye–Waller factor previously reported [29]. This difference may be due to the different anneal parameters used here (250 °C for 60 s), which differ from the 250 °C for 30 min typically used to optimize emission intensity [53], potentially leading to a higher level of broadband background emission in the observed range.

Reflection spectra of bullseye and bowtie cavities resonant with the ZPL and a bullseye cavity resonant with the FPR are displayed in Figure 3(b). The quality factor of the cavities is determined by fitting a Fano lineshape to the reflection curves [54] (see Section 5 of the Supplementary information). For bullseye cavities, *Q* ≈ 186; for bowtie cavities, *Q* ≈ 104. The difference between the measured and simulated quality factors can be attributed to fabrication imperfections such as surface roughness and a slightly slanted sidewall profile. These imperfections appear to have a more significant effect for the bowtie cavities as compared to the bullseye cavities. We observed a small redshift (≈3 nm) in the cavity resonance for the bowtie compared to that of the bullseye (see Figure S6 in the Supplementary information), as predicted by the simulation.

**Figure 4:**
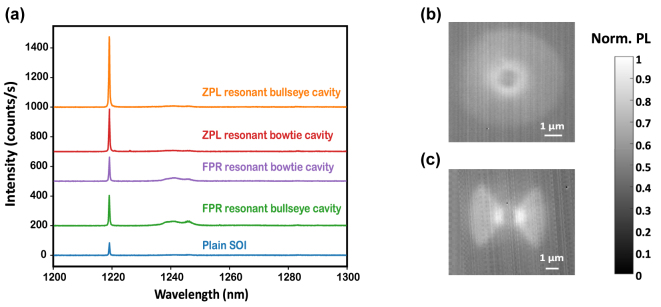
W center photoluminescence (PL) enhancement in resonant cavities. (a) PL spectra showing the zero-phonon-line (ZPL) enhancement in the bullseye and bowtie cavities (orange and red curves respectively), as well as first-phonon-replica (FPR) enhancement (green and violet, respectively), compared to the emission from W centers in unpatterned silicon-on-insulator (blue curve). (b) A near-field, near-infrared image of the ZPL resonant bullseye cavity when pumped with a 635 nm continuous-wave laser in the center of the cavity. The annular shape shows strong PL scattering from the first few rings of the cavity. (c) Same as (b) for a bowtie cavity.

Figure 4(a) shows the emission spectrum at 10 K for W centers in all these cavities and in unpatterned SOI. For the ZPL-resonant bullseye cavity, we observed an enhancement of the ZPL intensity by a factor of ≈5.5 compared to W centers in unpatterned SOI, giving a DW factor of ≈75% . For the ZPL-resonant bowtie, we observed an enhancement of the ZPL intensity by a factor of ≈3.5 , giving a DW factor of ≈60% . The PL enhancement of the ZPL resonant bowtie cavities is thus comparable to that of the ZPL resonant bullseye cavities and could be further improved by increasing the cavity enclosure angle *θ*. For the FPR-resonant cavities, we observed a small PL enhancement in the FPR.

Figure 4(b and c) show the near-field imaging of PL from the ZPL resonant bullseye and bowtie cavities captured by the infrared camera. The annular emission profile of the bullseye cavity suggests a strong scattering of emitted light from the first trench of the cavity. For the resonant bowtie cavity, we observe two lobes of emission coming from the first trenches of the cavity.

**Figure 5:**
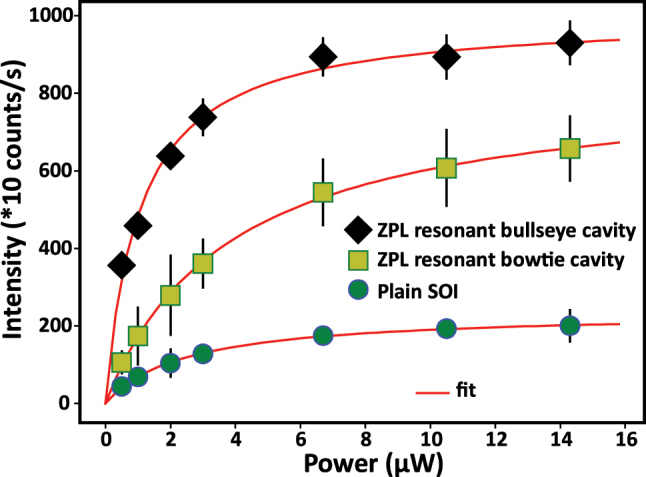
Zero-phonon-line (ZPL) intensity as a function of pump power for bullseye cavity resonant with the ZPL (black diamond markers), bowtie cavity resonant with the ZPL (yellow squares), and unpatterned (plain) silicon on insulator (SOI) (green circles) under 1 MHz pulsed laser excitation.

Figure 5 shows the PL intensity as a function of excitation laser power for the ZPL resonant bullseye cavity, ZPL resonant bowtie cavity, and unpatterned SOI. The data are fitted with the saturation function for a two-level system, *I*_sat_/((1 + *P*_sat_/*P*)). The fitted saturation power, *P*_sat_ = (1.1 ± 0.2) μW, *P*_sat_ = (3.8 ± 0.4) μW, and *P*_sat_ = (2.5 ± 0.2) μW, respectively, for the ZPL resonant bullseye cavity, ZPL resonant bowtie cavity, and unpatterned SOI. We observe an irreversible PL intensity drop for the resonant bullseye cavity at laser fluences greater than 12 mJ/cm^2^, suggesting damage due to photoionization processes [55]; we note that anomalous saturation has also been observed for single W centers [17]. From the saturation curve fits, we observed a ZPL collection enhancement factor *α*_1_ ≈ 5 for the resonant bullseye cavity and *α*_1_ ≈ 3 for the resonant bowtie cavity compared to W centers in unpatterned SOI.

To estimate an upper bound in the enhancement of extraction efficiency (collection to the first lens), we considered the coupling efficiency *η*_2_ from the first lens to the single-mode PL collection fiber in our setup. This efficiency was estimated by calculating the overlap integral between the fiber mode and the cavity mode image projected onto the fiber facet by our fiber-collection lens of focal length *f* = 16 mm (see Figure S4 in the Supplementary information). To obtain the electric field profile of the projected cavity emission, we used finite-different time-domain calculations to simulate the far-field emission from the cavity mode and applied a low-pass spatial filter to emulate the limited NA of the objective. The filtered farfield was Fourier-transformed to produce a focused image with the magnification specified by our experimental setup. (Details of these calculations are provided in Section 6 of the Supporting information.) The same procedure was applied to estimate *η*_2_ for a dipole in unpatterned SOI (see Figure S7 in the Supplementary information). We can then estimate an upper bound for the ZPL extraction enhancement for an ensemble of W centers in resonant cavities, *η*_total,e_ = *α*_1_/*η*_2_. These results are shown in Table 1.

**Table 1:** Experimentally observed collection efficiency enhancement (*α*_1_) and inferred extraction efficiency (*η*_total,e_) for an ensemble of W centers in ZPL-resonant bowtie and bullseye cavities. *η*_2_ is the modal overlap efficiency for a single-mode fiber obtained from simulations (details in the Supplementary information). The simulated, single W center extraction efficiencies into a 0.9 NA objective (*η*_1_) and collection efficiencies (*η*_total,c_) into a single-mode fiber represent theoretical upper bounds.

	Experimental			Theoretical	
Hosting geometry	*α*_1_	*η*_2_	*η*_total,e_ = *α*_1_/*η*_2_	*η*_1_	*η*_total,c_ = *η*_1_ × *η*_2_
Unpatterned SOI	–	≈32%	–	≈2.5%	≈1%
ZPL-resonant bowtie	≈3×	≈15%	≈6×	≈28%	≈4%
ZPL-resonant bullseye	≈5×	≈15%	≈11×	≈45%	≈7%

For comparison, Table 1 also presents calculated extraction efficiencies into a 0.9 NA collection lens (*η*_1_) and subsequent collection into a single-mode optical fiber (*η*_total,c_ = *η*_1_ × *η*_2_). To determine these efficiencies, we first calculate the extraction efficiency and the efficiency of emission into the cavity mode, *β*, for a single W center optimally placed at the center of the geometries. We then calculate the average $\bar{\beta }$ for W centers distributed throughout the cavity. (Calculation details are given in Section 9 of the Supplementary information.) The average extraction efficiency is then taken to be reduced from the single-emitter extraction efficiency by the factor $\beta /\bar{\beta }$ .

### 5.2 Measurement of Purcell enhancement

**Figure 6:**
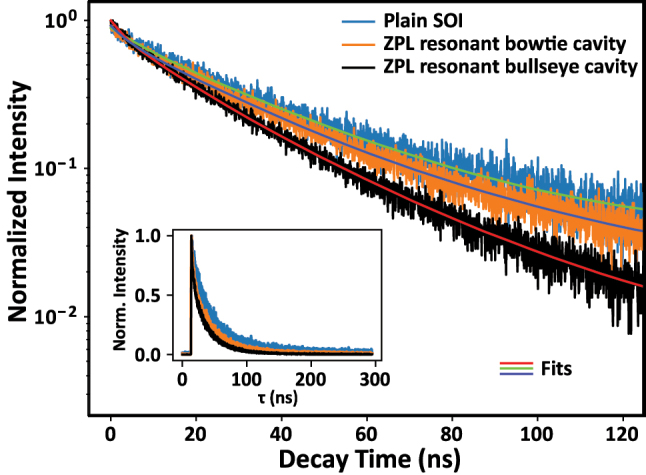
Time-resolved photoluminescence (PL) decay trace. PL zero-phonon line (ZPL) decay traces for W center ensembles in unpatterned silicon on insulator and bullseye and bowtie cavities resonant with the ZPL. Faster decays are apparent for the two cavities. Inset: same curves plotted on a linear scale for the first 300 ns. Curve fit on the first 120 ns of the lifetime trace using a biexponential decay function.

W centers were excited with a 640 nm pulsed laser at a repetition rate of 1 MHz to perform time-resolved PL measurements. The laser pulses had a temporal pulse width of ≈100 ps. Decay lifetime measurements were performed in the linear power-dependent regime of the emitters well below the saturation power. Figure 6 shows the lifetime traces of the W centers in unpatterned SOI and in ZPL-resonant bullseye and bowtie cavities for a pump power of 0.5 μW measured before the objective. The faster decay of the emitters in the cavity compared to the bulk lifetime suggests Purcell enhancement. Multi-exponential decay is apparent in all traces. To quantify the decay rate, the lifetime traces of the first 120 ns were fitted with a biexponential function plus a constant background, *A*_1_ exp(−*t*/*t*_1_) + *A*_2_ exp(−*t*/*t*_2_) + *B*. The first few nanoseconds in the time trace were ignored to remove background luminescence from silicon and potential laser leakage contributions (see Figure S8(A) in the Supplementary information).

We observed long (*t*_1_) and short (*t*_2_) lifetime components in all three cases, as tabulated in Table 2. The long lifetime component of the bulk W center is close to the monoexponential decay time of the W center ensemble reported previously [53]. The shorter lifetime component, which has not previously been reported, may come from some other background emission created in the same spectral range of the W centers or from interactions within the dense ensemble of defects. The estimated Purcell factors are based only on the long lifetime components, *t*_1_, corresponding to more than 70 % of the photon counts. Dividing the W center ensemble lifetimes in ZPL-resonant bullseye and bowtie cavities by the lifetime in unpatterned SOI, we obtain enhancement factors of 1.26 ± 0.07 and 1.08 ± 0.06, respectively.

**Table 2:** Biexponential fitting parameters for the photoluminescence decay traces in Figure 6, for W center ensembles emitting in unpatterned silicon on insulator and bullseye and bowtie cavities resonant with the zero-phonon line. The parameters *t*_1_ and *t*_2_ are, respectively, the slow and fast decay times and *A*_1_ and *A*_2_ are the corresponding weights.

	*A*_1_	*t*_1_	*A*_2_	*t*_2_
Plain SOI	0.73 ± 0.04	(32.8 ± 1.7) ns	0.13 ± 0.04	(7.3 ± 2.6) ns
ZPL resonant bullseye	0.76 ± 0.02	(26.1 ± 0.62) ns	0.17 ± 0.02	(5.7 ± 0.8) ns
ZPL resonant bowtie	0.7 ± 0.02	(30.3 ± 0.7) ns	0.16 ± 0.02	(4.4 ± 0.7) ns

We have also performed PL decay measurements on cavities that are not resonant with the ZPL. We have observed faster decay rates for W centers in FPR-resonant bullseye and bowtie cavities, in comparison with W centers in plain SOI (see Figure S8(B) in the Supplementary information). This suggests that the small PL intensity enhancement observed in the spectrum for FPR-resonant cavities (Figure 4) is due to both increased extraction efficiency and Purcell enhancement provided by the cavity.

We also observed a small dependence of decay rate on excitation power while collecting ZPL and PSB photons for W centers in unpatterned SOI and in the cavities (see Figure S9 in the Supplementary information). This can be attributed to local heating by the excitation laser. Such behavior is consistent with that reported for W centers in silicon embedded in a fully etched resonant bullseye cavity [41]. However, the effect was considerably less pronounced in our cavities with partially etched gratings, indicating more efficient heat transfer out of the cavities.

## 6 Discussion and conclusions

We have designed and demonstrated circular Bragg grating cavities on standard silicon photonic SOI wafers containing ensembles of W-type color centers produced by carbon ion implantation. The cavities were designed to improve the extraction efficiency of photons emitted by the W centers, which is otherwise very low due to silicon’s relatively large refractive index. We have observed an enhancement of zero-phonon-line emission intensity by a factor of ≈5 , together with a photoluminescence decay lifetime reduction by a factor of ≈1.3 . These observations indicate a Purcell enhancement of part of the emitter ensemble combined with increased extraction efficiency by the resonant cavity. We give a quantitative estimation of the collection efficiency by calculating the modal overlap with the collection fiber and infer an enhancement of ZPL extraction of ≈11 times. Bowtie cavities exhibit comparable extraction efficiency and Purcell enhancement to the bullseye cavities. Our work thus paves the way toward using bowtie cavities to develop chip-scale bright quantum light sources because they enable in-plane excitation both optically and electrically.

Our work differs from previous work that involved W centers in CBG cavities [41], [50] in various relevant aspects. First, we have etched the silicon device layer partway instead of all the way to the underlying oxide layer, providing higher extraction efficiency and efficient thermal conduction. Second, in addition to investigating cavities with full circular symmetry (bullseye cavities), we have also investigated bowtie cavities with broken circular symmetry. Since the cavity center can remain connected to the Si device layer, bowtie cavities feature efficient thermal dissipation regardless of partial or full etching of the gratings, and they can allow access from the sides for waveguide-based optical excitation or current injection. Lastly, while the creation of W centers for quantum photonic devices has been primarily investigated through Si implantation, we show that implantation with carbon ions can also be employed and may offer advantages in specific scenarios.

Although we have observed enhanced radiative rates and photon extraction consistent with the Purcell effect, significantly higher values could be achieved by selectively placing single W centers in the center of a resonant bullseye cavity with its dipole moment aligned with the polarization of the antinode of the cavity field [43], [47], [56], [57], [58]; calculations indicate that Purcell factors of 4.5 and extraction efficiencies of 65 % should be achievable. Selectively placing single W centers in the center of bowtie cavities with a large enclosure angle can also help us attain the figure of merits promised by bullseye cavities with additional benefits, including in-plane excitation and efficient thermal dissipation. Moreover, strategies that involve minimal lattice damage in silicon to create single color centers, such as focused-ion beam implantation [59] or laser annealing [55], are also promising routes to obtain efficient coupling of single emitters to microcavities and achieve high Purcell factors and efficient single-photon emission. We anticipate that such a capability will allow a better understanding of competing cavity coupling and nonradiative effects toward achieving color-center-based quantum light sources with near-unity DW factor and quantum efficiency.

## Supplementary Material

Supplementary Material Details
