# Supplementary material for: Enhanced zero-phonon line emission from an ensemble of W centers in circular and bowtie Bragg grating cavities
Source: Nanophotonics. 2024 Nov 19;14(11):1939–48. doi: 10.1515/nanoph-2024-0485 (PMC12133313; doi:10.1515/nanoph-2024-0485)
Supplement: Supplementary file 1 — Supplementary Material Details [file j_nanoph-2024-0485_suppl_001.pdf]

Enhanced zero-phonon line emission from an ensemble of W centers in circular and bowtie Bragg grating cavities Supplementary Information

Vijin Kizhake Veetil[1], Junyeob Song[2,3], Pradeep N. Namboodiri[2], Nikki Ebadollahi[4], Ashish Chanana[2,3], Aaron M. Katzenmeyer[4], Christian Pederson[2,4], Joshua M. Pomeroy[2], Jeffrey Chiles[5], Jeffrey Shainline[5], Kartik Srinivasan[2,6], Marcelo Davanco[2], and Matthew Pelton[1]

[1] Department of Physics, University of Maryland Baltimore County, 1000 Hilltop Circle, MD 21250, USA
[2] National Institute of Standards and Technology, 100 Bureau Dr, Gaithersburg, MD 20899, USA
[3]Theiss Research La Jolla, CA 92073, USA
[4]University of Maryland College Park, 4500 Campus Dr, College Park, MD 20742, USA
[5] National Institute of Standards and Technology, 325 Broadway, Boulder, CO 80305, USA
[6]Joint Quantum Institute, 4254 Stadium Dr, College Park, MD 20742, USA

1 Finite-element modeling for cavity optimization

We selected the optimized cavity parameters, Λ=440 nm, w=90 nm, b= 350 nm, t_{trench}= 140 nm, t_{slab} = 80 nm, where Λ is the period of the grating, w is the trench width, b is the grating width, t_{trench} is the trench depth and t_{slab} is the thickness of the unetched silicon layer to get good spectral matching with the ZPL of W centers. These parameters give an extraction efficiency of ≈ 65 % for a single W center located in the center of a circular cavity. The trench depth was optimized by performing a series of simulations with different ratios t_{slab}/t_{trench}, as shown in **Figure S1(A)**. The resonance wavelength blueshifts by more than 30 nm as the trench depth increases from 120 nm to 220 nm (fully etched device layer). Moreover, the full-width half maximum (FWHM) of the resonance peak becomes narrower when the trench depth increases, which indicates stronger field confinement and, consequently, a higher Purcell factor. For a fully etched bullseye cavity, by contrast, we calculate an extraction efficiency (η) of ≈ 45 %, which indicates that more than half of the light is lost into the bottom dielectric stack. In addition to partial etching, other strategies to improve η are to fabricate a suspended structure by etching away the buried-oxide layer [1, 2] or/and to use a bottom gold layer [3–5] that acts as a mirror to reflect light into the upper dielectric space.

Figure S1(B) shows calculated Purcell factors (F_p) for different t_{slab}/t_{trench} ratios. F_p close to 12 is observed for a fully etched bullseye cavity, although it has the lowest extraction efficiency. The bullseye cavity with t_{trench}= 170 nm, t_{slab} = 50 nm has a relatively high F_p and η, and is thus selected as the optimized device structure.

Figure S2(A,B) shows the variation of extraction efficiency (η) and Purcell factor (F_p), respectively, in a 0.9 NA objective for bowtie cavities at different cavity enclosure angles.

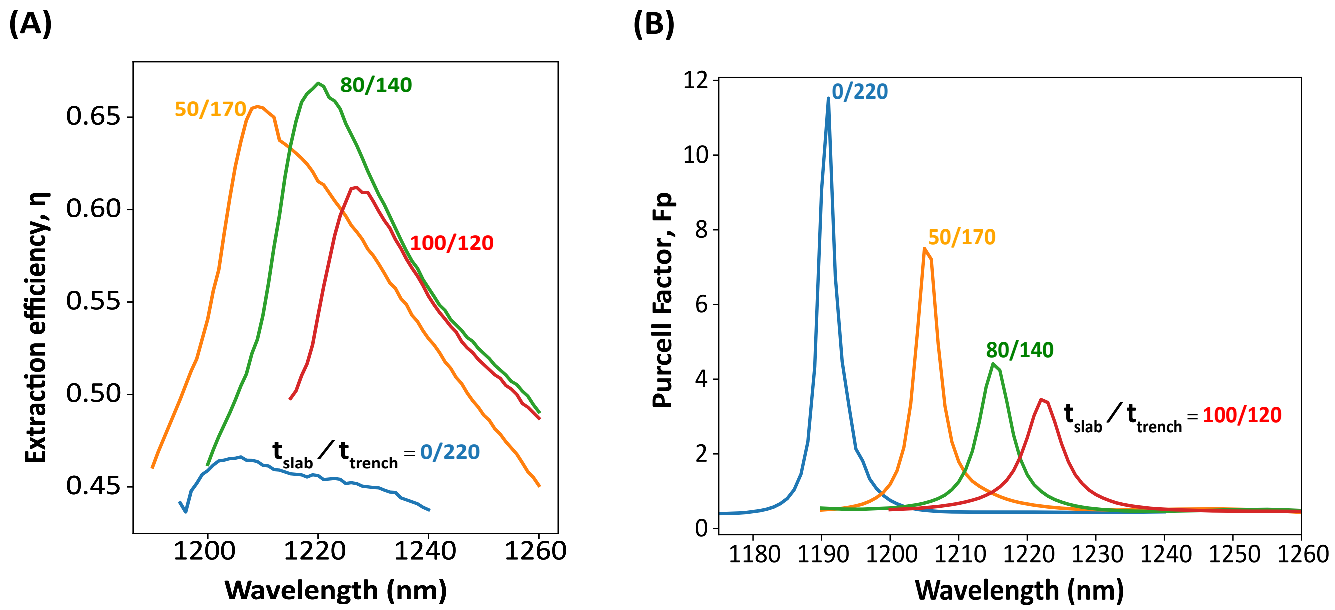

Figure S1: Partial etching of the bullseye cavity (A) The variation of extraction efficiency (η) in a 0.9 NA objective for different $\frac{t_{slab}}{t_{trench}}$ ratios.(B) The variation of Purcell factor, F_p, for different $\frac{t_{slab}}{t_{trench}}$ ratios. Changing the $\frac{t_{slab}}{t_{trench}}$ ratio shifts the cavity resonance, which can be reset by changing the cavity pitch.

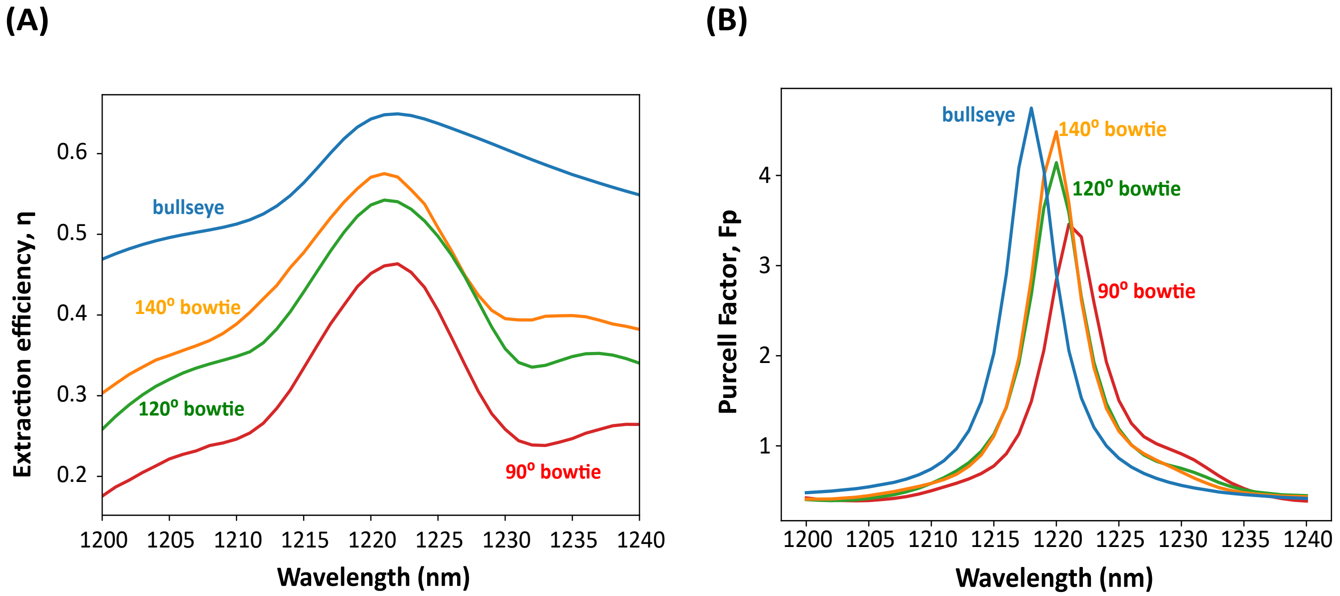

Figure S2: Bowtie cavities with t_{slab}/t_{trench} ratio = 80/140 at different cavity enclosure angle (A) The variation of extraction efficiency (η) in a 0.9 NA objective for bowtie cavities with a cavity enclosure angle of 90°, 120° and 140° (B) The variation of Purcell factor, F_p, for the same variation of cavity enclosure angles. η and F_p of a bullseye cavity are also given for reference.

2 Stopping and Range of Ions in Matter (SRIM) simulations of carbon implantation

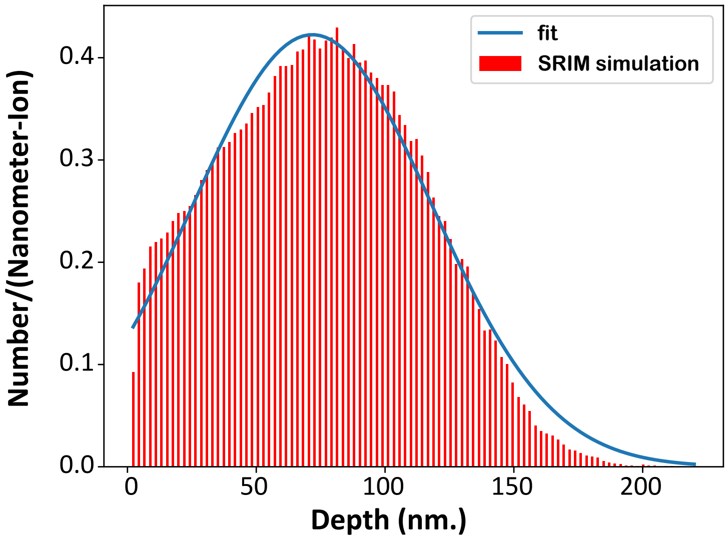

Figure S3: SRIM simulation [6] plot showing the vacancy distribution generated by implanting 30 keV carbon ion into silicon. W centers produced by implanting carbon are assumed to follow this distribution curve. The vacancies generated act as a potential well that captures the interstitial silicon created by carbon implantation to form W centers.

3 Cavity fabrication

For the fabrication of a circular Bragg grating cavity on a silicon-on-insulator (SOI) substrate, we employed electron-beam (e-beam) lithography combined with inductively coupled plasma-reactive ion etching (ICP-RIE). The SOI substrate, featuring a device layer of thickness 220 nm, was initially subjected to sonication in acetone and isopropyl alcohol (IPA) and subsequently rinsed with deionized water. This was followed by applying a positive e-beam resist, which was spin-coated onto the substrate and then baked. To mitigate charging effects during the e-beam lithography, a charge dissipation layer was applied and baked. The e-beam writing process was then executed, after which the sample was rinsed with deionized water, developed using hexyl acetate, and rinsed with IPA again. After a plasma residue cleaning, the grating structure was etched using an ICP-RIE employing the pseudo-Bosch process. This process uses sulfur hexafluoride (SF_6) and octafluorocyclobutane (C_4F_8) gases to achieve a vertical sidewall profile. Finally, the etch process mask was removed using a resist stripper and the substrate was given a final rinse with IPA.

4 Experimental setup

Figure S4 shows the schematic of the measurement setup used in this work. The experimental methods section of the main article gives an overview of the experimental components used in the optical measurements. Here, we give some additional details about the instruments for completeness.

We used a 4 K closed-cycle cryostat with an internal 100×, 0.9 numerical aperture (NA) objective accessible from the top of the sample chamber, through which the sample is illuminated and photoluminescence (PL) is collected. An XYZ nanopositioner stack provides a three-axis range of motion for the sample in the cold and under vacuum with respect to the internal fixed objective. Vacuum-compatible grease was used to ensure thermal contact between the sample and the stage. We used a fiber-coupled diode laser source at a wavelength of 635 nm for continous-wave (CW) pumping of color centers. The emission spectrum was obtained by sending the PL to a 0.5 m spectrograph equipped with a liquid nitrogen-cooled one-dimensional 1024-pixel array detector. A grating density of 1000 lines/mm at a center wavelength of 1.3 μm and a 100 μm slit width was used, giving a resolution of $\approx$ 130 pm. A short-wave infrared camera with a 2D InGaAs TE-cooled detector array was used to perform near-field imaging of bullseye cavities. A picosecond diode laser at 640 nm wavelength was used for pulsed excitation at 1 MHz repetition rate for lifetime measurements. The output PL was sent to an superconducting nanowire single photon detector with an efficiency of $\approx$ 80 % and was connected to a two-channel time-correlated single photon counting (TCSPC) system to perform time-resolved PL measurements. For measurements of the cavity resonances, we used the reflectivity of a supercontinuum laser. The supercontinuum was generated by combined pumping at 1064 nm and its second harmonic at 532 nm in a highly nonlinear fiber, so a notch filter at 1064 nm was used to sufficiently attenuate the pump light.

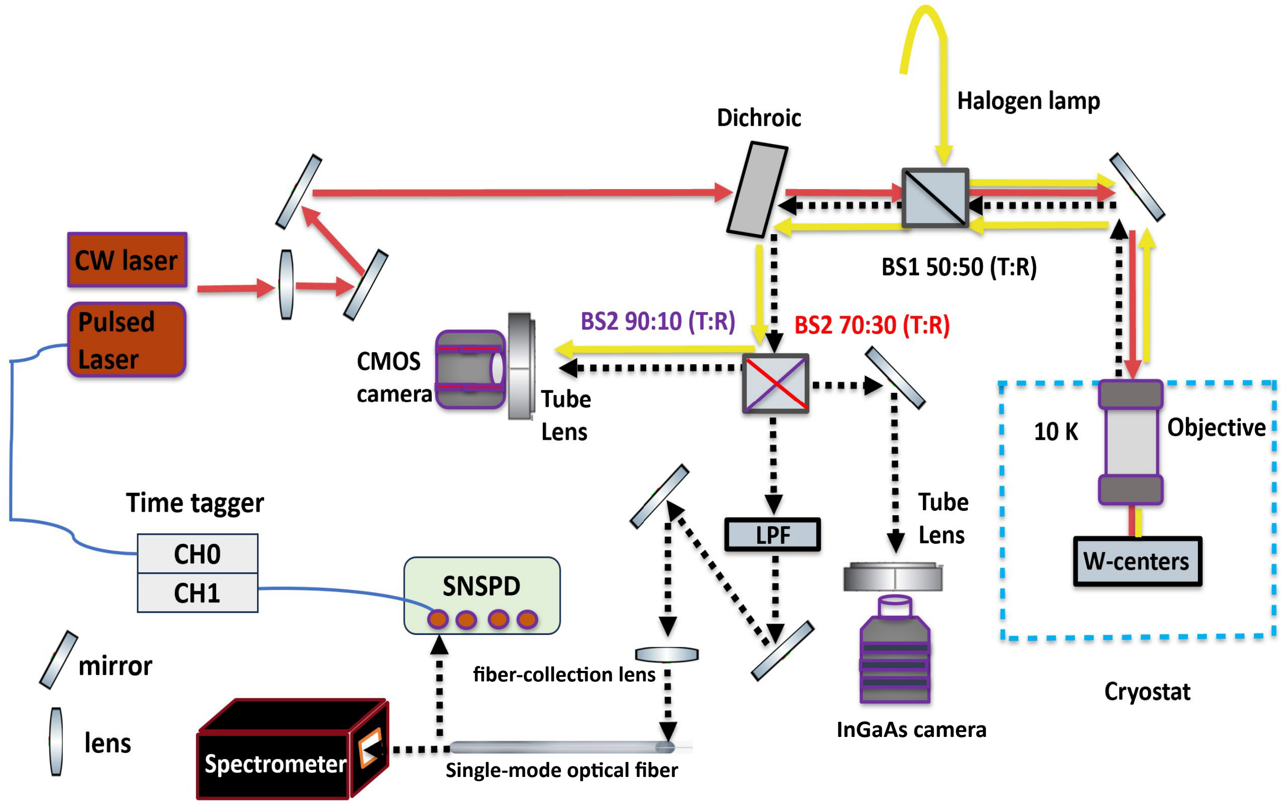

Figure S4: Schematic of the experimental setup. LPF: long pass filter, BS: Beamsplitter, T: Transmission, R: Reflection, CHO: Channel 0, CH1: Channel 1, SNSPD: Superconducting Nanowire Single-Photon Detector.

5 Cavity quality factor estimation

The asymmetric lineshape observed for the cavity reflection measurements can be attributed to Fano-like interference between the continuum background of light directly reflected from the cavity and the light that is reflected after being resonantly coupled to the cavity [7]. To estimate the quality factor (Q) of the resonant bullseye and bowtie cavities, the reflection spectrum is fitted with a Fano line shape

$$I(\omega) = A_0 \frac{(p\Gamma/2 + (\omega - \omega_c))^2}{(\Gamma/2)^2 + (\omega - \omega_c))^2} + B_0 + C_0 * \omega^2 \tag{1}$$

where ω_0 is the center frequency of the cavity mode, Γ is the cavity resonance linewidth; A_0, B_0, and C_0 are constants and the quadratic background comes from interference of light reflected from planar surfaces in the SOI stack. The ratio between the resonant and non-resonant scattering amplitudes is described by the parameter p, called the Fano parameter. Note that when $p = 0$, the Fano lineshape converges to a Lorentzian function, corresponding to only resonant reflection from the cavity mode. We use equation 1 to fit the reflectivity curves to obtain ω_c and Γ. The quality factor from the fit parameters is calculated as $Q = \omega_c/\Gamma$.

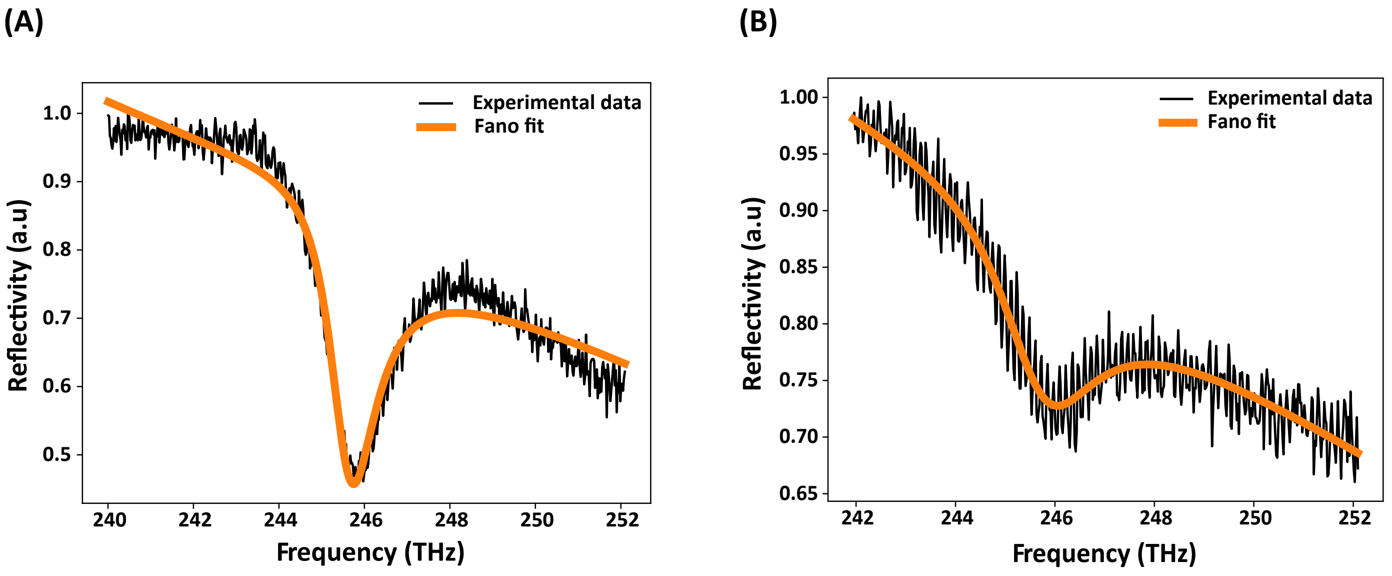

Figure S5: Cavity quality factor estimation by fitting with a Fano lineshape (A) Fano fit on ZPL resonant bullseye cavity reflectivity curve. (B) Same for ZPL resonant bowtie cavity.

Figure S5(A, B) shows the reflectivity curves of ZPL-resonant bullseye and ZPL-resonant bowtie cavities fitted with the respective Fano lineshapes. From the fits, we obtain $\omega_c = 245.59 \pm 0.03$ and $\Gamma = 1.32 \pm 0.06$ for the bullseye cavity corresponding to a Q of 186 ± 8.45. For the resonant bowtie cavities, $\omega_c = 245.68 \pm 0.14$ and $\Gamma = 2.37 \pm 0.24$ corresponding to a Q of 104 ± 10.50.

6 Bowtie-cavity reflection spectra

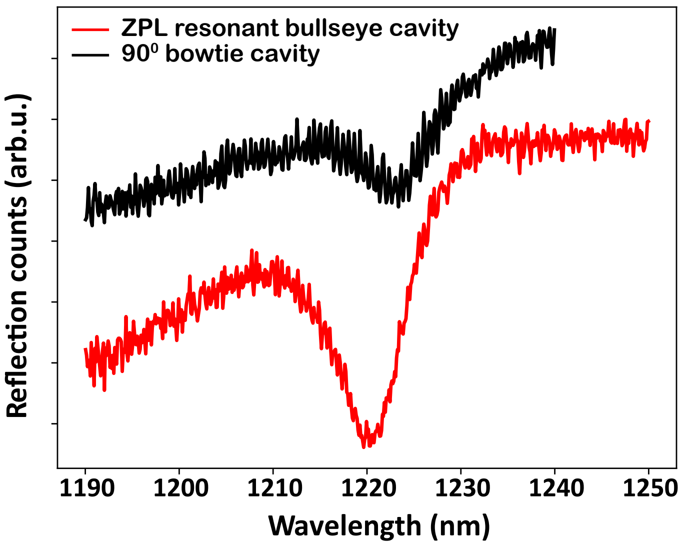

Figure S6: Reflectance spectra of ZPL resonant bullseye cavity (red curve) and its corresponding 90° bowtie cavity (black curve) showing an ≈ 3.5 nm redshift in resonance wavelength. The reflection spectra are normalized to the reflection counts from the gold markers on the sample surface.

7 Collection efficiency estimation

Finite Difference Time Domain (FDTD) simulations were performed to obtain the image of the cavity mode produced by the optical system onto the photoluminescence collection fiber facet. As seen in the schematic of Fig. S4, such an image is produced by focusing light coming from the objective onto the collection fiber facet with a fiber-collection lens. In the FDTD simulation, an electric point dipole was placed at the center of the cavity and allowed to radiate with a short temporal envelope. The produced electromagnetic field was allowed to evolve over a sufficiently long time scale to encompass only the cavity resonance. A steady-state electromagnetic (modal) field was recorded over a surface located 200 nm above the cavity surface and was subsequently used to obtain a vector far-field projection. The unit vector components from the far-field projection were used to filter out the waves corresponding to the 0.9 NA acceptance angle of the objective. The resulting electric field components were then Fourier transformed to produce the final projected image. The simulation was set up with the cavity or unpatterned SOI lying in the x-y plane and a W center dipole oriented at $\theta = \pi/4$, $\phi = \pi/4$, where θ and ϕ are the polar and azimuthal angles in spherical coordinates. **Figure S7(A)** shows the electric field intensity profile obtained for a W center dipole oscillating at the ZPL frequency in a ZPL resonant bullseye cavity and **Figure S7(B)** shows the electric field intensity profile obtained for a W center dipole oscillating at the ZPL frequency in bulk SOI. We use SMF-28 fiber for collection; the fundamental mode of this fiber at 1218 nm is shown in **Figure S7(C)**. The overlap integral between these matrices is calculated numerically to obtain the modal overlap efficiency (η_2) using the expression in equation 2.

$$\eta_2 = \frac{|\iiint_V \sum_{i=x,y,z} E_{1,i}E^*_{2,i}\, dx\, dy\, dz\,|^2}{(\iiint_V \sum_{i=x,y,z} E_{1,i}E^*_{1,i}\, dx\, dy\, dz)(\iiint_V \sum_{i=x,y,z} E_{2,i}E^*_{2,i}\, dx\, dy\, dz)} \tag{2}$$

where, E_1 is the cavity electric field and E_2 is the fiber field. Experimentally, the measured counts in the detector are maximized by aligning the collection fiber. Consequently, to achieve the modal overlap between the cavity's near-field and the optical fiber in the calculations, one of the dual lobes of the electric field distribution observed in the image plane of the cavity is scanned across the fiber mode until maximum overlap is obtained.

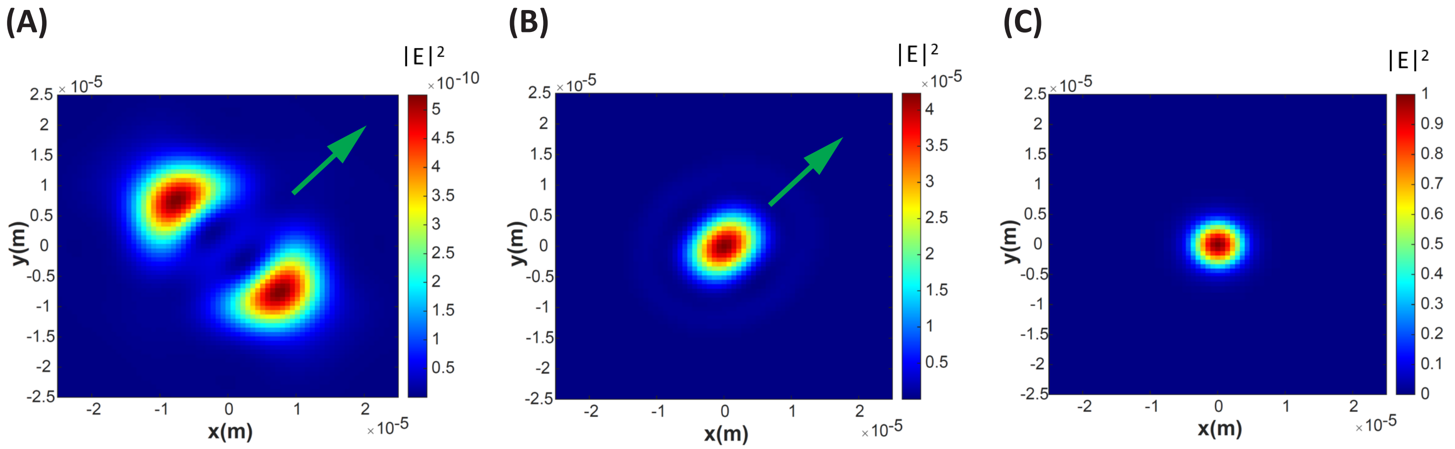

Figure S7: Electric field distribution on the image plane for a W center dipole in a resonant cavity and bulk SOI. A green arrow indicates the dipole moment orientation. (A) W center dipole in a ZPL resonant cavity. (B) W center dipole in bulk SOI. (C) Fundamental mode for an SMF-28 fiber at 1218 nm.

8 Lifetime curve fitting, trace clipping, and FPR cavity lifetime

Figure S8(A) shows the PL lifetime traces of the unpatterned SOI, the ZPL-resonant cavity, and the background emission from the SOI substrate (outside the implant box). The laser profile is added to the plot to illustrate the temporal pulse duration of the laser used for excitation. The shaded region in the plot has been discarded while fitting the lifetime curves for the W centers in the cavities to remove the contribution of the background light.
Fig. S8(B) shows PL lifetime traces for FPR-resonant bullseye and bowtie cavities.

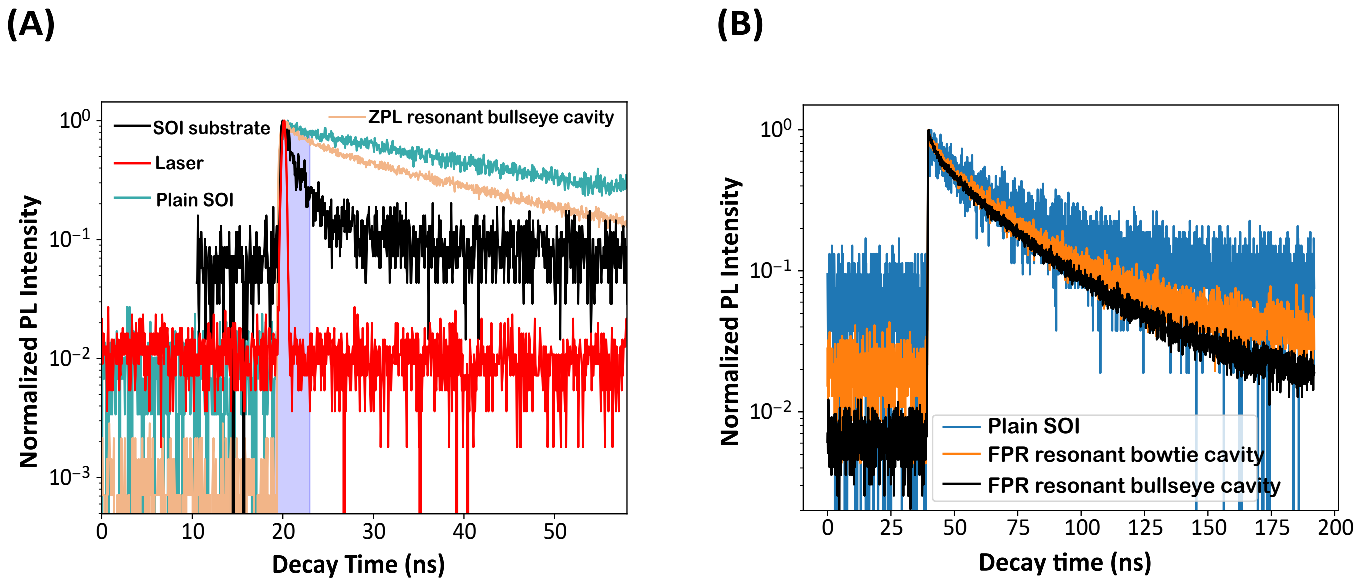

Figure S8: PL decay curve of ZPL photons under 1 MHz pulsed laser excitation at 0.5 μW pump power. (A) Lifetime trace of W centers in plain SOI (cyan curve), W centers in ZPL resonant bullseye (peach curve), and background emission from SOI (black curve). The red curve represents the temporal profile of the pulsed laser. The blue-shaded region highlights the lifetime trace clipped to remove contributions from laser and background emission (B) Lifetime trace of FPR photons in an FPR resonant bullseye (black curve) and bowtie (orange curve) cavity indicating a faster decay compared to plain SOI (blue curve).

9 Power dependant decay rate

We performed lifetime fitting by collecting only ZPL photons from the W centers. We made measurements for multiple excitation laser powers (**Fig. S9(A,B)**) and did not notice a considerable change in the decay rate. However, while collecting both the ZPL and PSB photons, we noticed the decay rate is sensitive to the excitation laser power, as shown in **Fig. S9(C,D)**. This behavior was previously reported for W centers and is attributed to the local heating caused by increasing laser power.

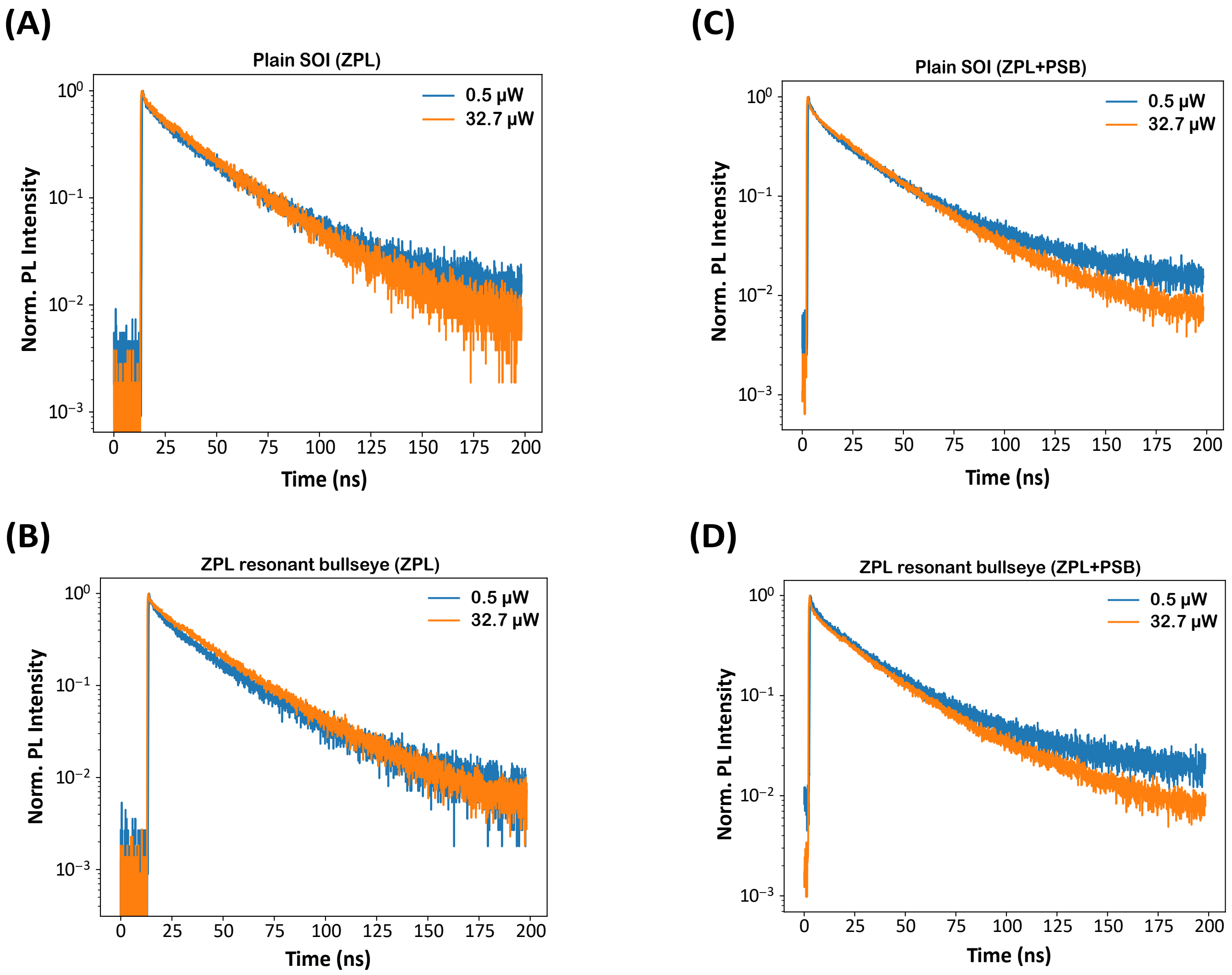

Figure S9: Power dependent decay rate. (A, B) PL decay trace of ZPL photons for bulk W centers and ZPL resonant bullseye at laser powers 0.5 μW and 32.7 μW. No significant decay acceleration was observed at these two laser powers while collecting only the ZPL. (C, D) PL decay trace of ZPL + PSB photons of the same cavities for the same laser powers shows a small PL decay acceleration.

10 Effective mode volume (V_{eff}) and average beta factor ($\overline{\beta}$) estimation

The effective mode volume, $V_{eff} = 1.45(\lambda_c/n)^3$ of the bullseye cavity is calculated using the expression

$$V_{eff} = \frac{\int \epsilon(r) \mid E(r) \mid^2 \ d^3r}{max(\epsilon(r) \mid E(r) \mid^2)}, \tag{3}$$

where $\epsilon(r)$ is the permittivity of the medium and E(r) is the modal electric field. Equation 3 indicates that the V_{eff} is the ratio of the total energy stored in the cavity volume to the maximum energy density. The maximum energy density for a bullseye cavity is located in the center of the bullseye cavity, where the electric field is at its maximum.

Since the quality factor of a cavity mode, Q $\approx$ 203, and $V_{eff} = 1.45(\lambda_c/n)^3$, the maximum Purcell factor is given by

$$F_{p,max} = \frac{3Q(\frac{\lambda_c}{n})^3}{4\pi^2 V_{eff}} \approx 11 \tag{4}$$

For a quantum emitter at a position r in the cavity, the Purcell factor is given by the following expression

$$F_p(r) = F_{p,max}\frac{|\mathbf{p}\cdot\mathbf{E(r)}|^2}{|p|^2|E_{max}|^2}f(\Delta) \tag{5}$$

where p is the dipole moment of the quantum emitter, E(r) is the cavity field at any location r, E_{max} is the cavity field in the center, and f(Δ) is the spectral detuning between the emitter frequency and the cavity mode frequency. It can be noted that equation 5 is consistent with equation 4 when the dipole is ideally coupled to the resonant mode.

W center dipoles take two orientations relative to the crystal axes, [110] and [1 $\bar{1}$0] in the wafer plane after projecting the [111] dipole moment onto the wafer surface [8]. We these two directions to correspond to the x and y axes. Moreover, the z component of the dipole can also be coupled to the weak TM component of the cavity field. Therefore, $F_p(r)$ for the resonant bullseye cavity can be decomposed into three terms corresponding to a dipole coupled to the E_x component, E_y component, and E_z component of the mode field:

$$F_p(r) = F_{p,max}\frac{|E_x^2|\cos^2(\pi/4) + |E_y^2|\cos^2(\pi/4) + 2|E_z^2|\sin^2(\pi/4)}{|E_{max}^2|} \tag{6}$$

Note that we have also assumed that the factor due to spectral detuning, f(Δ)=1, which is the case here since the emitter ensemble PL linewidth (FWHM $\approx$ 0.3 nm) is much narrower than the cavity resonant mode (FWHM $\approx$ 8 nm). In addition to the two dominant resonant modes, the cavity supports a continuum of leaky modes. The ratio of the radiative transition rate into the leaky modes over the radiative transition rate in bulk silicon (Γ_o) is defined as γ, and the ratio between the radiative transition rates in the cavity and in the bulk silicon is F_p. For a ZPL resonant bullseye cavity, ZPL photons are emitted into the resonant cavity mode at a rate of $F_p\Gamma_o$ and into the leaky modes at a rate of $\gamma\Gamma_o$. Therefore, the fraction of photons emitted into the cavity mode is given by $\beta = F_p/(F_p + \gamma)$. For a single dipole in the center of the cavity at 45° with respect to the cavity plane, $F_p = 4.5$, $\gamma = 0.4$, and hence $\beta \approx 0.92$. γ is estimated by fitting the Purcell factor curve from the simulation of a single dipole placed in the center of a bullseye cavity. The Purcell factor for a large detuning from the resonance wavelength estimates the decay rate into the leaky modes, and we have assumed it to be constant for all the dipoles in the cylindrical cavity volume.

For the ensemble of W centers, the average β factor can be estimated using the $F_p(r)$ expression from equation 6 and integrating it over a distribution of W centers in a cylindrical volume defined by the diffraction-limited laser spot size ($\approx$ 400 nm). A local XY PL scan in the implant box shows a uniform emission intensity, indicating a homogeneous density of W centers in the plane. However, the spatial distribution of the W centers across the silicon thickness depends on the implantation parameters. Since the W centers are generated by carbon implantation in silicon, we assume the silicon interstitials are captured by the vacancies produced during the implantation to form W centers. The W center distribution $\rho(r)$ in the z direction is obtained by fitting the vacancy distribution curve generated by Stopping and Range of Ions in Matter (SRIM) simulations [6] in **Fig. S3**. Thus,

$$\overline{\beta} = \frac{\iiint_V \rho(r)(\frac{F_p(r)}{(F_p(r)+0.4)}dV}{\iiint_V \rho(r)dV} \approx 0.64 \tag{7}$$

11 Uncertainty reporting

The uncertainties reported in the main text for the simulated Q factor (Section 2), W-center ensemble photoluminescence saturation power (Section 5.1) and radiative decay lifetimes (Section 5.2) were obtained from fits (type A evaluation), and correspond to 95 % confidence intervals (two standard deviations). Uncertainties for the radiative decay enhancement factors are obtained by propagating the lifetime uncertainties.

In Fig. 5 of the main text, each point in the scatter plot is calculated by taking the mean of 10 bins of 100 ms bin width, and the uncertainty bars are shown for two standard deviations.

References

[1] Marcelo Davanco, Matthew T Rakher, Dieter Schuh, Antonio Badolato, and Kartik Srinivasan. A circular dielectric grating for vertical extraction of single quantum dot emission. *Applied Physics Letters*, 99(4), 2011.

[2] Sascha Kolatschek, Stefan Hepp, Marc Sartison, Michael Jetter, Peter Michler, and Simone Luca Portalupi. Deterministic fabrication of circular bragg gratings coupled to single quantum emitters via the combination of in-situ optical lithography and electron-beam lithography. *Journal of Applied Physics*, 125(4), 2019.

[3] Andrea Barbiero, Jan Huwer, Joanna Skiba-Szymanska, David JP Ellis, R Mark Stevenson, Tina Muller, Ginny Shooter, Lucy E Goff, David A Ritchie, and Andrew J Shields. High-performance single-photon sources at telecom wavelength based on broadband hybrid circular bragg gratings. *ACS Photonics*, 9(9):3060–3066, 2022.

[4] Reza Hekmati, John P Hadden, Annie Mathew, Samuel G Bishop, Stephen A Lynch, and Anthony J Bennett. Bullseye dielectric cavities for photon collection from a surface-mounted quantum-light-emitter. *Scientific Reports*, 13(1):5316, 2023.

[5] Lucas Rickert, Timm Kupko, Sven Rodt, Stephan Reitzenstein, and Tobias Heindel. Optimized designs for telecom-wavelength quantum light sources based on hybrid circular bragg gratings. *Optics Express*, 27(25):36824–36837, 2019.

[6] James F Ziegler. Srim-2003. *Nuclear instruments and methods in physics research section B: Beam interactions with materials and atoms*, 219:1027–1036, 2004.

[7] Matteo Galli, SL Portalupi, Michele Belotti, LC Andreani, Liam O'Faolain, and TF Krauss. Light scattering and fano resonances in high-q photonic crystal nanocavities. *Applied Physics Letters*, 94(7), 2009.

[8] Yoann Baron, Alrik Durand, Tobias Herzig, Mario Khoury, Sébastien Pezzagna, Jan Meijer, Isabelle Robert-Philip, Marco Abbarchi, Jean-Michel Hartmann, Shay Reboh, et al. Single g centers in silicon fabricated by co-implantation with carbon and proton. *Applied Physics Letters*, 121(8), 2022.
